# Propofol Induces Postoperative Depression and Inhibits Microglial Function in Mice

**DOI:** 10.1155/2019/7651383

**Published:** 2019-06-10

**Authors:** Feng Song, Xiao Lv, Jing Meng

**Affiliations:** ^1^Department of Orthopedics, Qingdao Municipal Hospital, Qingdao, Shandong, China; ^2^Department of Cardiac Surgery, Qingdao Municipal Hospital, Qingdao, Shandong, China; ^3^Department of Spinal Surgery, Qingdao Municipal Hospital, Qingdao, Shandong, China

## Abstract

Many patients experience excellent physical recoveries after surgery; however, there are some of them who from suffer mood fluctuation, even depression. Postoperative depression may be resulted from cognitive dysfunction, pain, and a compromised immune system during the surgery. But there is a higher possibility that general anaesthesia may be responsible for the development of depression. Here, we employed one of the most used anaesthetics, propofol, in a mouse model to investigate whether this intravenous anaesthetic compound could cause depressive-like behavioural performance in mice. We found a single dose of propofol caused significant abnormal behavioural performance in tail suspension, forced swimming, and open field tests. We also examined the brain section of these mice and revealed that there was significant reduced expression of the CD11b protein, which demonstrated an inhibition of propofol on microglial function. We investigated the effect of propofol on synaptic protein, SYP, and found there was no notable influence on the protein expression. These above results suggested that propofol treatment might promote the depressive-like behaviours in mice via influencing the microglial cell function. Furthermore, we found the level of the IL-6 cytokine was significantly increased in the brain tissue, which might subsequently cause the activation of the transcriptional factor, STAT3. Our finding may provide a new perspective of further understanding the mechanism of anaesthetic drugs and deciphering the underlying mechanism of postoperative depression.

## 1. Introduction

Many patients who undergo general anaesthesia or surgery experience some form of postsurgical depression, especially during the six months following an invasive procedure [1]. As one of the frequent complications after surgery, depression may lead to further morbidity and mortality, especially for elderly patients [1]. Meanwhile, researchers have discovered that depressed patients are more likely to have other complications after surgery [2]. They are less able to cooperate well with the caregivers in their after-surgery care, such as rehabilitative therapy. In patients who already have a preexisting depression or anxiety history, the recovery time from postsurgical depression is much longer [3]. A study also suggested surgery might exacerbate the severity of the preexisting depression [4]. So far, little is known about why there is such a strong link between surgery and depression. Some researchers have thought the psychological reason per se is that many people experience postsurgical depression because the surgical procedures force them to confront their own mortality. Some studies also emphasized the specific types of surgery per se on the induction of postsurgical depression since depression seems to be more often observed in some major surgeries, including brain surgery, hip replacement surgery, and cancer resection. However, recent studies suggested that the length of time spent under anaesthesia seemed to be related to the likelihood and severity of depression [5]. Propofol is one of the most used anaesthetics in the intensive care setting after surgery [6]. Propofol treatment has been found to be significantly associated with cognitive dysfunctions in the postoperative period [7]. There is no effort made to examine whether and how propofol could impact the mood status in patients and animals so far. Therefore, we employed a mouse model with a single dose of propofol treatment and tested the depressive-like behaviours in the mouse model. We found mice exposed to a single dose of propofol treatment exerted anxiety-like behaviours in an open field test by showing less time in the center area and depressive-like behaviours in tail suspension and forced swimming tests by showing longer immobility time than control mice without injection of propofol. Our results indicated that propofol might be responsible for the mood fluctuation after surgery and this effect may be associated with the influence of propofol on the microglia cells in the central nervous system (CNS).

## 2. Materials and Methods

### 2.1. Animals and Drug Treatment

In the present study, we used 8-10 weeks old male C57BL/6 mice. All mice were free to access water and food. For all the animal studies here, mice were treated strictly following the guidelines established by the Chinese Council on Animal Care. All the procedures were approved by the Animal Care Committee of Qingdao Municipal Hospital, Shandong, China. There were two groups of mice in the study: normal saline (controls; *n* = 10) and propofol (75 mg/kg; *n* = 10). A single-dose injection of propofol was administrated to the mice intraperitoneally. Commercial propofol injection solution (Xi'an Libang Pharmaceuticals, China) was used here. The dose of propofol used here is adapted from the previous study [8]. During the anaesthesia time, all mice were put on the heating pad to maintain their body temperature. Behavioural tests were performed 1 week later.

### 2.2. Open Field

A square wood box was used here for the open field test as previously described [9]. At the beginning of the test, each mouse was placed in a corner of the box facing the wall. The total traveled distance and total time spent in the inner squares of all mice were recorded and measured in a 5-minute session.

### 2.3. Tail Suspension Test

For the tail suspension test, the procedures were performed as previously reported [10]. Mice were suspended through tails with a tape on a small metal hook, and they cannot escape or hold on to nearby surfaces in this position. The total time the mice spent immobile during the 6 min testing period was recorded. Immobility is defined as a lack of attempt of mice to move their bodies.

### 2.4. Forced Swimming Test

We carried out the forced swimming test as previously reported [11]. Mice were placed in a glass beaker filled with water at room temperature. We tested the total immobile time of mice in a 15 min testing session, and the last 6 min was scored for immobility duration. At the end of each test, the wet mice were immediately placed in a cage with normal bedding after they were warmed up in a dry towel.

### 2.5. Elevated Plus Maze Test

The elevated plus maze is a simple method for evaluating animal anxiety responses. We tested the anxiety level of mice by using the elevated plus maze assay as previously reported [12]. Basically, the elevated plus maze apparatus is equipped with two open and two closed arms and elevated to a height of around 50 cm above the ground. At the beginning of each test, the mouse was placed in the central square by facing the open arm and then was allowed to explore the arms for 5 min. The amount of time spent in the open arms was recorded and analyzed by a person who was not involved in the experimental design.

### 2.6. Enzyme-Linked Immunosorbent Assay (ELISA)

We measured the IL-6 level in brain tissues by using a commercial ELISA kit (eBioscience, Thermo Fisher Scientific). Each sample was assayed in a duplicate manner with appropriate dilutions in order that relative luminescent units could fall within the linear range of standard curves. The value of IL-6 from each sample was normalized and expressed as a ratio compared to the total loading protein as a relative ratio. The absorbance of each sample was measured with a microplate reader (Synergy Mx, BioTek, Winooski, VT).

### 2.7. Western Blot

Dissolved brain samples were processed and run on SDS-PAGE gels. They were then transferred onto PVDF membranes that were then blocked with 5% skim milk in TBST buffer. The blocked membranes were further investigated with antibodies to CD11b (1 : 4000; Abcam, UK), synaptophysin (SYP), p-Stat-3 (1 : 1000; Cell Signaling, Danvers, MA), and total Stat-3 (1 : 1000; Cell Signaling, Danvers, MA) in TBST milk overnight at 4°C. After incubation with the secondary antibodies for 2 hours at room temperature respectively, the bands of protein on the membrane were disclosed with chemiluminescence reaction. *β*-Actin was used as an internal control (1 : 5000; Santa Cruz Biotechnology, CA, USA). Quantitative results were expressed as a ratio of each target protein to its *β*-actin.

### 2.8. Statistical Analysis

Values presented in the study were shown as the mean ± SEM. The significance of difference between two groups was determined by Student's *t*-test analysis. A *p* value of less than 0.05 was regarded as statistically significant.

## 3. Results

### 3.1. Single Dose of Propofol Exposure Caused Depressive-Like Behaviours in Mice with Tail Suspension and Forced Swimming Tests

**Figure 1:**
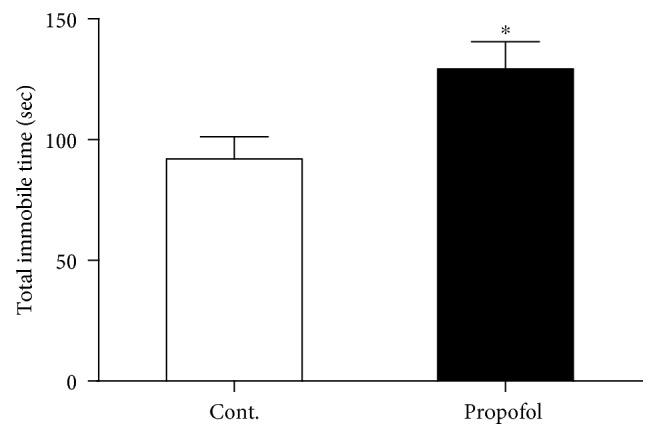
Propofol increased the total immobile time of mice compared to control mice in the tail suspension test. All data are expressed as the means ± SEM. *n* = 10. ^∗^*p* < 0.05.

**Figure 2:**
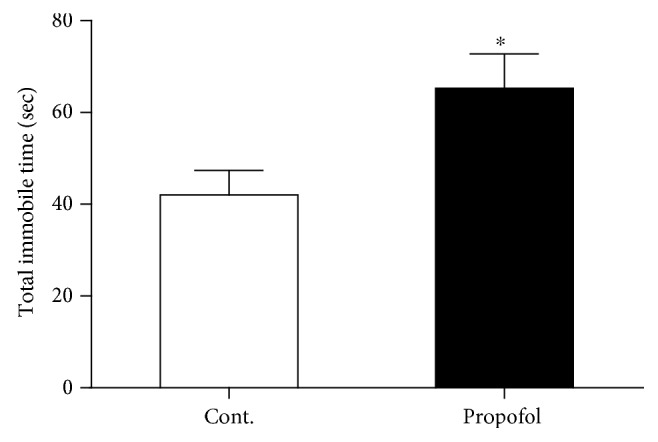
Propofol increased the total immobile time of mice compared to control mice in the forced swimming test. All data are expressed as the means ± SEM. *n* = 10. ^∗^*p* < 0.05.

Firstly, we tested the hypothesis whether a single dose of propofol treatment could cause long-term effects on the behavioural performance in mice by focusing on the depression manner. We employed the tail suspension and forced swimming tests in these mice to explore the long-term effects of propofol. In the tail suspension test, we found propofol significantly increased the immobile time compared to the control mice without propofol treatment when the mice were hanged in tails (Figure 1). To further confirm the depressive-like behaviours in the mice exposed to propofol, we used the force swimming method to measure the immobile time of these mice. Our results demonstrated that propofol increased the total time of immobile time compared to mice in the control group (Figure 2). These above findings revealed that a single dose of propofol treatment could cause and sustain the long-term depressive behavioural performance in mice.

### 3.2. Single Dose of Propofol Exposure Caused Anxiety-Like Behaviours in Mice with Open Field and Elevated Plus Maze Tests

**Figure 3:**
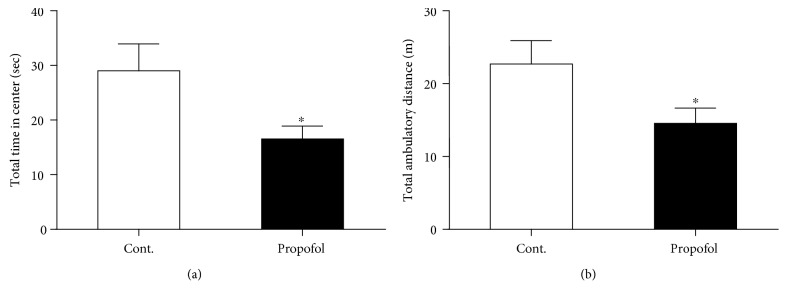
Propofol caused anxiety-like behaviours of mice in the open field test. (a) Propofol decreased the total time in the center area of mice compared to control mice in the open field test. (b) Propofol decreased the total travel distance of mice compared to control mice in the open field test. All data are expressed as the means ± SEM. *n* = 10. ^∗^*p* < 0.05.

**Figure 4:**
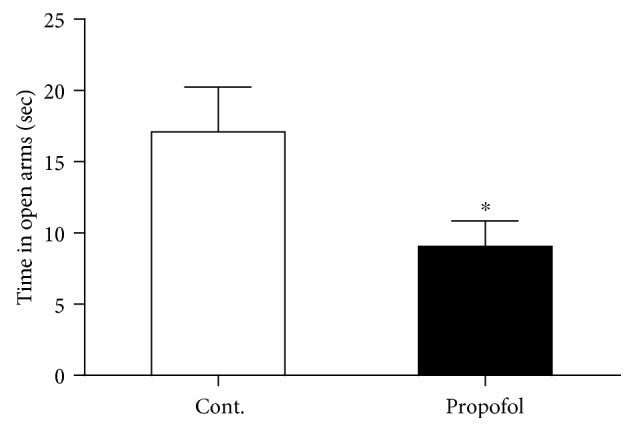
Propofol decreased the total time in the open arms of mice compared to control mice in the elevated plus maze test. All data are expressed as the means ± SEM. *n* = 10. ^∗^*p* < 0.05.

Anxiety is the highest cooccurrence complication in depression. Therefore, we postulated whether propofol could induce anxiety-like behaviours in these mice, basing on the above behavioural results in these mice. We used the open field assay to test the anxiety level of these mice. As shown in Figure 3(a), propofol treatment effectively reduced the total time mice spent on the center area in the open field. And we also measured another important parameter of the test, the total travel distance of the mice. As we expected, propofol injection significantly decreased the travel distance of mice in the 5 min test session compared to control mice (Figure 3(b)). Next, we investigated the anxiety-like behaviours of these mice in elevated plus maze assay. We found that mice exposed to propofol showed significant less time spent in open arms (Figure 4), which was in line with the results from the open field test.

### 3.3. Single Dose of Propofol Exposure Caused the Increased Level of IL-6 in the Brain Tissues of Mice

**Figure 5:**
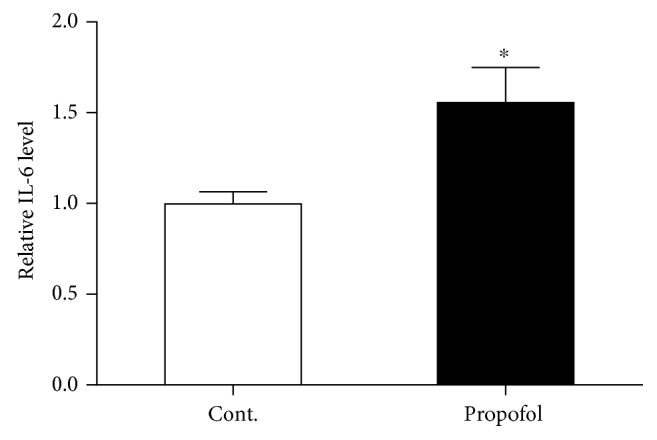
Propofol increased the IL-6 level in the brain tissues of mice compared to control mice. All data are expressed as the means ± SEM. *n* = 6. ^∗^*p* < 0.05.

A recent study suggested the involvement of neuroinflammation in postoperative delirium-like cognitive deficits [13]. We tested whether propofol could impact the expression level of IL-6 in the brain tissues of these mice. To achieve the conclusion, we performed ELISA assay in the brain tissues (hippocampus and cortex) to assess the level of IL-6 in mice with or without propofol treatment. Our results demonstrated that propofol treatment in this condition could be able to upregulate the expression level of this cytokine in the brains of mice exposed to propofol (Figure 5). This result indicated that propofol might cause the anxiety- and depressive-like behaviours in mice by affecting the inflammatory response in their brains.

### 3.4. Single Dose of Propofol Exposure Caused the Reduced Expression of CD11b and Increased Expression of p-STAT-3 in the Brain Tissues of Mice

**Figure 6:**
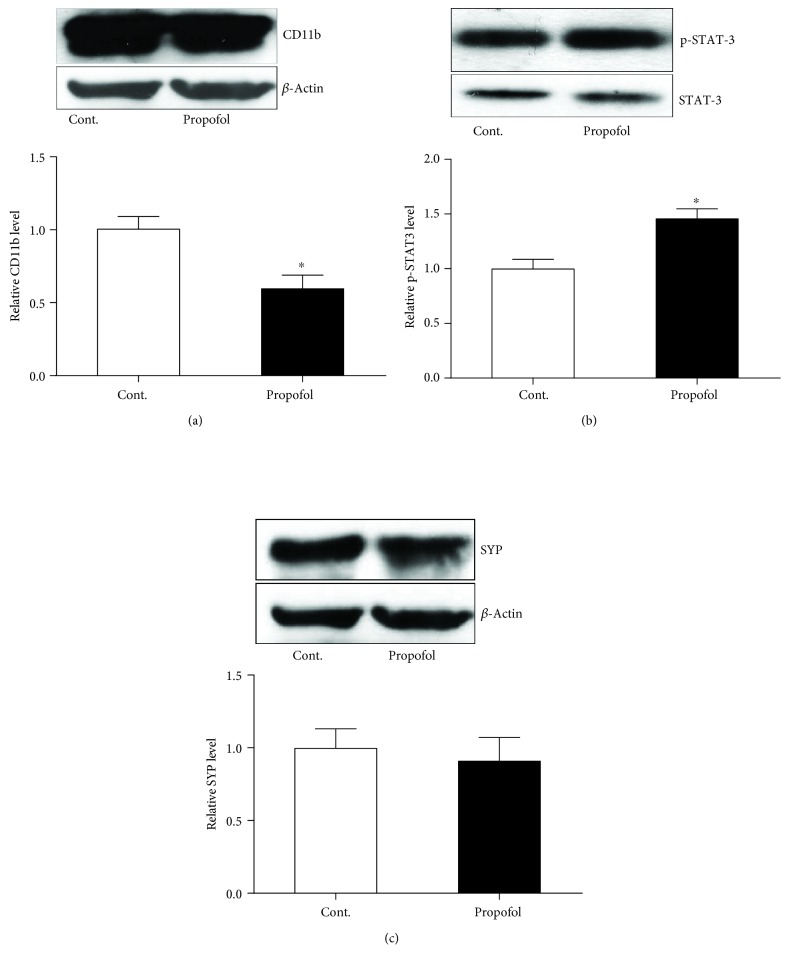
Propofol influenced the function of astrocytes and STAT-3 in the brain tissues of mice compared to control mice. (a) Propofol decreased the expression of the CD11b protein in the brain of mice. (b) Propofol increased the expression of the p-STAT-3 protein in the brain of mice. (c) Propofol did not change the expression of the SYP protein in the brain of mice. All data are expressed as the means ± SEM. *n* = 5. ^∗^*p* < 0.05.

Last, we probed the possible cellular and molecular mechanisms that might be responsible for the behavioural changes in these mice exposed to propofol. We tested whether glial cells were influenced by the propofol treatment by looking at the microglial maker protein, CD11b. With a western blot study, we found the expression level of CD11b was reduced in the brains of mice with propofol treatment (Figure 6(a)), which suggested that microglial function might be regulated by the propofol treatment. Meanwhile, the expression of the presynaptic protein SYP was not affected by the propofol treatment (Figure 6(c)). We also studied the function of the transcriptional factor STAT-3 by investigating the phosphorylation status of STAT-3 (p-STAT-3). Our western blot results demonstrated that propofol treatment caused the increased expression level of p-STAT-3 without affecting the expression of total STAT-3 (Figure 6(b)). These findings implicated that propofol might influence the microglial cell function and enhance the phosphorylation of STAT-3 while inducing the anxiety- and depressive-like behavioural performances in mice.

## 4. Discussion

Cognitive and memory dysfunctions have been fairly studied, but the postoperative mood fluctuation has not attracted enough attention so far. Some evidences supported the idea that there were significant mood changes that occurred in the patients who are exposed to surgery and anaesthetic treatment. Here, we tested a new hypothesis that anaesthetic treatment per se may be enough to significantly affect the mood status in animals that underwent the single dose of anaesthetic treatment without an accompanying surgical procedure. We used propofol as the representative anaesthetic compound and intraperitoneally injected it to the mice. In the following days, a series of depressive and anxiety behaviours were performed to observe the mood changes in the mice. We found that there were significant differences on the behavioural performances between these mice with or without propofol exposure. Since these propofol-induced effects were sustained for a significant time while even after the medication was took off, we postulated that these effects were not the acute anaesthetic influence but were mediated by other systems in the CNS.

Neuroinflammation has been found to be one of the major factors that contribute to the depression and other mood changes in the CNS [14]. Here, we asked whether a microglial cell was activated in the brain of mice after propofol exposure. The microglial cell protein marker, CD11b that is one of the common marker proteins to demonstrate the activation of microglia, was investigated with western blot. Surprisingly, our results indicated that propofol could inhibit the protein level of CD11b. The plausible explanation here is propofol could inhibit the CD11b protein expression regardless of whether the microglial cells were activated or not. The prominent advancement of propofol may be important for the potential treatment of alleviating the microglial overactivation in some pathological conditions of the CNS, such as trauma, stroke, and multiple sclerosis. We also looked into the effect of propofol on the synaptic proteins that are very important fundamental factors behind the behavioural performances of animals [15]. Interestingly, although propofol could affect the microglial cell function, it seemed not to impact the synaptic protein expression level. These findings are not in line with some previous reports [16]. The possible reason may be due to the difference of time window when the proteins were collected after the propofol was given. But our findings still suggested that propofol might have a different impact on neuron and other glial cells. In the future study, the effects of propofol on glial and neuronal cells should be elaborated individually.

The neuroinflammatory response in the CNS exposed to the single dose of propofol was further validated by the ELISA test of IL-6 in these two groups of mice. We found propofol treatment could induce an upregulation of IL-6 in the mouse brain. These findings were in line with the results of propofol on the microglial cell function. While inhibiting the CD11b component, propofol may activate the relevant signaling pathways that are important for the microglial cell activation.

Collectively, our study suggested propofol was able to influence the microglial cell function and exacerbate inflammatory response in the CNS. The cellular and molecular reaction might be responsible for the behavioural changes in these mice that are administrated with the single dose of propofol. Our study emphasized the important role of anaesthetics on the impact of mood changes after operation, but mood could be significantly impacted by many factors, including inflammation [17] that is quite often seen in the patients during and after surgery. Therefore, a series of well-designed, systemic studies on an animal model which involved not only the anaesthetics but also the surgery procedure are warranted for the future.

## Data Availability

The data used to support the findings of this study are available from the corresponding author upon request.
